# KBG syndrome: a case report of a 12-year-old male child with ADHD

**DOI:** 10.1192/j.eurpsy.2025.1097

**Published:** 2025-08-26

**Authors:** G. Lorenzo Chapatte, C. Alario Ruiz, L. Rodríguez Andrés, C. Rodríguez Valbuena, M. S. Geijo Uribe, M. Ríos Vaquero, L. Rojas Vázquez, A. Monllor Lazarraga, L. Sobrino Conde, M. Fernández Lozano, N. Navarro Barriga, B. Rodríguez Rodríguez, M. J. Mateos Sexmero, A. Aparicio Parras, M. P. Pando Fernández, P. Martínez Gimeno, M. A. Andreo Vidal, M. Calvo Valcárcel, M. D. L. Á. Guillén Soto, L. del Canto Martínez, F. J. González Zapatero, M. E. Espinosa Muth

**Affiliations:** 1 Psychiatry; 2 Psychology, Hospital Clínico Unversitario de Valladolid, Valladolid, Spain

## Abstract

**Introduction:**

KBG syndrome is a rare genetic condition (autosomal dominant inheritance - ANKRD11 mutation) with a particular phenotype (short stature, craniofacial dysmorphism and skeletal abnormalities) and neurodevelopmental delay or intellectual disability. Patients with this condition usually have a greater tendency to present symptoms related to impulsivity and distractibility typical of ADHD, as well as behavioral disturbances and aggressiveness.

We present the case of a 12-year-old male child who is being followed up at the Child and Adolescent Mental Health Department for intellectual disability and ADHD combined.

**Objectives:**

To address the importance and multidisciplinary approach of the KBG Syndrome in the pediatric population with a diagnosis of ADHD based on the presentation of the aforementioned clinical case.

**Methods:**

Bibliographic search and description of a clinical case of a patient under follow-up for Child and Adolescent Mental Health at the “Hospital Clínico Universitario de Valladolid”.

**Results:**

A 12-year-old boy from Spain was referred to the Child and Adolescent Psychiatry Department for attention and concentration difficulties and impulsivity problems. The parents describe the patient as a restless and nervous child who is sometimes aggressive in moments of important frustration. They point out that he impulsively performs dangerous acts, such as crossing the road without making sure that a car is not coming by, and is easily distracted. The School Center reports that the child is incapable of following the rules and shows great difficulty in learning.

He has a medical history of growth delay with problems of right dorsal scoliosis requiring the use of a night corset, moderate hypoacusis in the right ear, myopia and astigmastism and macrodontia. He also underwent surgery for epigastric hernia.

In addition, he shows a particular phenotype that shares with his mother and for which genetic studies have been performed that determined the ANKRD11 mutation, confirming the diagnosis of KBG syndrome.

**Conclusions:**

KBG syndrome is a rare genetic condition that should be considered in the differential diagnosis of patients with cognitive and behavioral difficulties in combination with a distinctive phenotype.

The importance of the diagnosis of this entity lies in being able to offer a better multidisciplinary medical approach at the organic and mental health level.

It is also important to be able to plan and propose an adapted education at school and to provide tools and management strategies to the family at home for a better prognosis and quality of life of the patient and his environment.

**Disclosure of Interest:**

None Declared

